# Beyond the amyloid−β framework: reconsidering the complementary role of tau-related mechanisms in sleep-cognition research

**DOI:** 10.3389/fneur.2026.1751087

**Published:** 2026-10-27

**Authors:** Ting Wu, Min Yin

**Affiliations:** 1 Department of Neurology, The First Affiliated Hospital with Nanjing Medical University, Nanjing, Jiangsu, China; 2 Department of Otorhinolaryngology, The First Affiliated Hospital with Nanjing Medical University, Nanjing, Jiangsu, China

**Keywords:** amyloid−β, glymphatic system, sleep and cognition, tau pathology, tau-related mechanisms

## 1 Introduction

Sleep disturbance is increasingly recognized as an important modifiable risk factor for cognitive decline and Alzheimer's disease (1, 2). Over the past decade, the amyloid−β (Aβ) hypothesis has provided a major mechanistic framework linking sleep disruption with neurodegeneration (3, 4). Numerous experimental and clinical studies have demonstrated bidirectional interactions between sleep disturbance and Aβ accumulation through mechanisms including impaired glymphatic clearance (5), altered neuronal activity, and disrupted protein homeostasis (6). These findings have substantially advanced our understanding of sleep-related neurodegeneration.

Despite these important advances, several observations suggest that the current framework may not fully explain all aspects of sleep-related cognitive dysfunction.

In this review, the term “Aβ-centric” refers to the predominant tendency to interpret sleep-related cognitive changes primarily through Aβ accumulation and clearance mechanisms. The result is a growing portfolio of Aβ-centric papers, trials, and funding streams, alongside a striking under-representation of tau-focused hypotheses. Recent data challenge this imbalance, revealing rapid, robust, and behaviorally relevant fluctuations in tau species after experimental sleep deprivation—effects that can be dissociated from parallel Aβ changes (7). It is therefore timely to ask whether the field's Aβ-centric default is obscuring alternative, and potentially more actionable, therapeutic avenues.

Importantly, this review does not aim to refute the role of Aβ in the long-term pathogenesis of Alzheimer's disease. Rather, our focus is on sleep-related, short-term cognitive changes and their underlying molecular dynamics, where emerging evidence suggests that tau-related mechanisms may play a more immediate and functionally relevant role. Clarifying this temporal distinction is essential to avoid overgeneralization of findings derived from acute experimental paradigms.

## 2 Established evidence supporting the Aβ framework

Aβ hypothesis has provided the dominant mechanistic framework linking sleep disturbance with cognitive decline. Experimental and clinical studies consistently demonstrate a bidirectional relationship in which sleep disruption promotes Aβ accumulation, while Aβ pathology further impairs sleep architecture, particularly slow-wave sleep (8). Moreover, the discovery of the glymphatic system has provided a physiological explanation for this interaction, showing that sleep facilitates Aβ clearance from the brain, whereas sleep loss compromises this process (9). Human PET imaging and cerebrospinal fluid biomarker studies further support the association between poor sleep, increased Aβ burden, and cognitive decline (10).

Collectively, these findings establish Aβ as a fundamental mechanism underlying sleep-related neurodegeneration. However, accumulating evidence suggests that Aβ alone may not fully explain all aspects of sleep-related cognitive dysfunction, particularly the rapid cognitive changes observed following acute sleep disruption, highlighting the need to consider complementary mechanisms such as tau pathology.

## 3 Remaining knowledge gaps beyond the established Aβ framework

### 3.1 Remaining causal questions

Although the amyloid−β (Aβ) framework has substantially advanced our understanding of sleep-related neurodegeneration, several questions regarding causality remain. Acute sleep deprivation can impair cognitive function within hours, whereas detectable changes in cerebral Aβ deposition generally occur over much longer time scales. In addition, although anti-Aβ therapies have demonstrated reductions in amyloid burden, their effects on cognitive outcomes have generally been modest. These observations do not diminish the importance of Aβ but suggest that additional mechanisms may contribute to the cognitive consequences of sleep disruption. Among these, tau-related pathways have emerged as a promising complementary mechanism that warrants further investigation, particularly in the early stages of sleep-related cognitive impairment (11).

### 3.2 Tau as a relatively underexplored mechanism

Acute sleep restriction can elevate CSF tau and phosphorylated-tau epitopes in proportion to EEG slow-wave loss. These tau increments correlate with next-day memory deficits even when Aβ concentrations remain stable (12). Nevertheless, such findings are typically relegated to secondary analyses or interpreted as secondary to Aβ modulation, may have contributed to comparatively less attention devoted to tau-related mechanisms in sleep research.

The rapid increase in CSF tau following sleep deprivation may reflect multiple physiological processes, including heightened neuronal activity, metabolic stress, or synaptic remodeling. Therefore, tau elevation should not be interpreted exclusively as a pathogenic signal. However, the consistent association between tau dynamics and next-day cognitive performance suggests that tau may function not only as a state marker but also as a potential mediator of sleep-related cognitive vulnerability. Further mechanistic studies are required to disentangle these possibilities.

### 3.3 Structural reinforcement of Aβ bias

Historically, technical limitations, including the broader availability of Aβ PET imaging compared with tau biomarkers, may have contributed to the predominance of Aβ-focused investigations (13). Tau verification still relies on lumbar puncture, higher costs, and lower throughput, creating a feedback loop that starves tau mechanistic studies of resources and visibility.

## 4 Implications of a Tau-inclusive perspective: missed therapeutic windows

Recognizing the potential contribution of tau-related mechanisms does not diminish the established importance of Aβ but may broaden current conceptual models linking sleep disturbance with cognitive decline. Integrating both pathways could facilitate a more comprehensive understanding of disease heterogeneity, particularly across different stages of cognitive impairment. However, current evidence supporting tau-directed interventions in sleep-related cognitive dysfunction remains preliminary, and robust longitudinal clinical studies are required before any therapeutic implications can be established.

### 4.1 Future research priorities for integrating tau into sleep-cognition research

Within the context of sleep-related and potentially early-stage cognitive changes, current evidence suggests that tau-related mechanisms deserve greater investigation alongside the well-established Aβ framework. Rather than replacing the existing paradigm, future studies may benefit from integrating both Aβ- and tau-related pathways to better characterize the temporal evolution of sleep-related cognitive dysfunction.

#### 4.1.1 Conceptual integration

Future research should consider sleep-related cognitive impairment as a multifactorial process in which Aβ− and tau-related mechanisms may contribute differently across disease stages. Such an integrated framework may better explain the heterogeneity observed in experimental and clinical studies than models focusing on either pathway alone.

#### 4.1.2 Research priorities

Longitudinal studies combining objective sleep assessments with multimodal biomarkers—including Aβ PET, tau PET, cerebrospinal fluid biomarkers, plasma phosphorylated tau, and neuroimaging—are needed to clarify the temporal relationships among sleep disturbance, protein pathology, and cognitive decline. Mechanistic studies should also evaluate whether tau changes mediate the cognitive effects of sleep disruption independently of, or in conjunction with, Aβ pathology.

#### 4.1.3 Translational considerations

Although emerging preclinical studies suggest that tau-targeted therapies may represent promising future strategies, evidence supporting their clinical benefit in sleep-related cognitive impairment remains limited. Future randomized clinical studies will be required to determine whether combining sleep-based interventions with disease-modifying therapies can improve cognitive outcomes beyond currently available approaches.

## 5 Conclusion

The amyloid−β (Aβ) framework has substantially advanced current understanding of the relationship between sleep disturbance and cognitive decline and remains central to the pathogenesis of Alzheimer's disease. At the same time, accumulating evidence suggests that tau-related mechanisms may represent an important complementary pathway, particularly in the early cognitive consequences of sleep disruption.

Rather than challenging the established Aβ paradigm, we propose that integrating Aβ− and tau-related mechanisms may provide a more comprehensive understanding of sleep-related cognitive dysfunction across different stages of disease progression. Importantly, the current evidence supporting tau-related mechanisms is still evolving, and many observations are derived from experimental or observational studies. Therefore, future longitudinal human studies incorporating multimodal biomarkers will be essential to determine the respective contributions of Aβ and tau and to establish their translational relevance.
